# Supplementary material for: Direct Photochemical Synthesis of Substituted Benzo[b]fluorenes
Source: Org Lett. 2024 Nov 25;26(48):10364–8. doi: 10.1021/acs.orglett.4c03978 (PMC11629382; doi:10.1021/acs.orglett.4c03978)
Supplement: Supplementary file 2 — ol4c03978_si_002.zip [file ol4c03978_si_002.zip › FID for Publication/20240913_RC-2-C-5-158-F30-39_01/plots/RC-2-C-5-158-F30-39_gHMBCAD_20240913_01_plot01.pdf]

RC-2-C-5-158-F30-39

Location **1**	Pulse sequence **gHMBCAD**	Temperature **25**	Suboperator **RuairiCrawford**
Date collected **2024-09-13**	Solvent **cdcl3**	Spectrometer **Agilent-NMR-vnmrs500**	Operator **MBaumann**

SAMPLE

date	Sep 13 2024
solvent	cdcl3
sample	20240913_RC-2-C-5-158-F30-39_01

ACQUISITION

sw	6157.6
at	0.150
np	1848
fb	4000
ss	32
d1	1.000
nt	16

2D ACQUISITION

sw1	30177.3
ni	400
phase	arrayed

PRESATURATION

satmode	n
wet	n

TRANSMITTER

tn	H1
sfrq	500.033
tof	48.8
tpwr	62
pw	7.000

DECOUPLER

dn	C13
dof	1285.9
dm	nnn
decwave	W40_One_NMR_new
dmf	32258
dpwr	39
pwxlvl	59
pwx	9.500

HMBC

j1xh	146.0
jnxh	8.0

ADIABATCIC

pwx180ad	One_NMR_new_ad30 0
pwxlvl180	52
pwx180	465.4

FLAGS

hs	nn
sspul	y
PFGflg	y
hsglvl	6408

SPECIAL

temp	25.0
gain	38
spin	0

GRADIENTS

gzlvl1	512
gt1	0.001000
gzlvl3	1536
gt3	0.001000
gstab	0.000500

F2 PROCESSING

sb	-0.075
sbs	not used
fn	2048

F1 PROCESSING

gf1	0.012
gfs1	not used
proc1	lp
fn1	4096

DISPLAY

sp	-529.8
wp	6157.6
sp1	-1886.7
wp1	30177.3
rfl	529.8
rfp	0
rfl1	1886.7
rfp1	0

PLOT

wc	216.8
sc	0
wc2	124.5
sc2	12.2
vs	108
th	2
ai cdc av	

ACQUISITION ARRAYS

array	phase
arraydim	800
i	phase
1	1
2	2

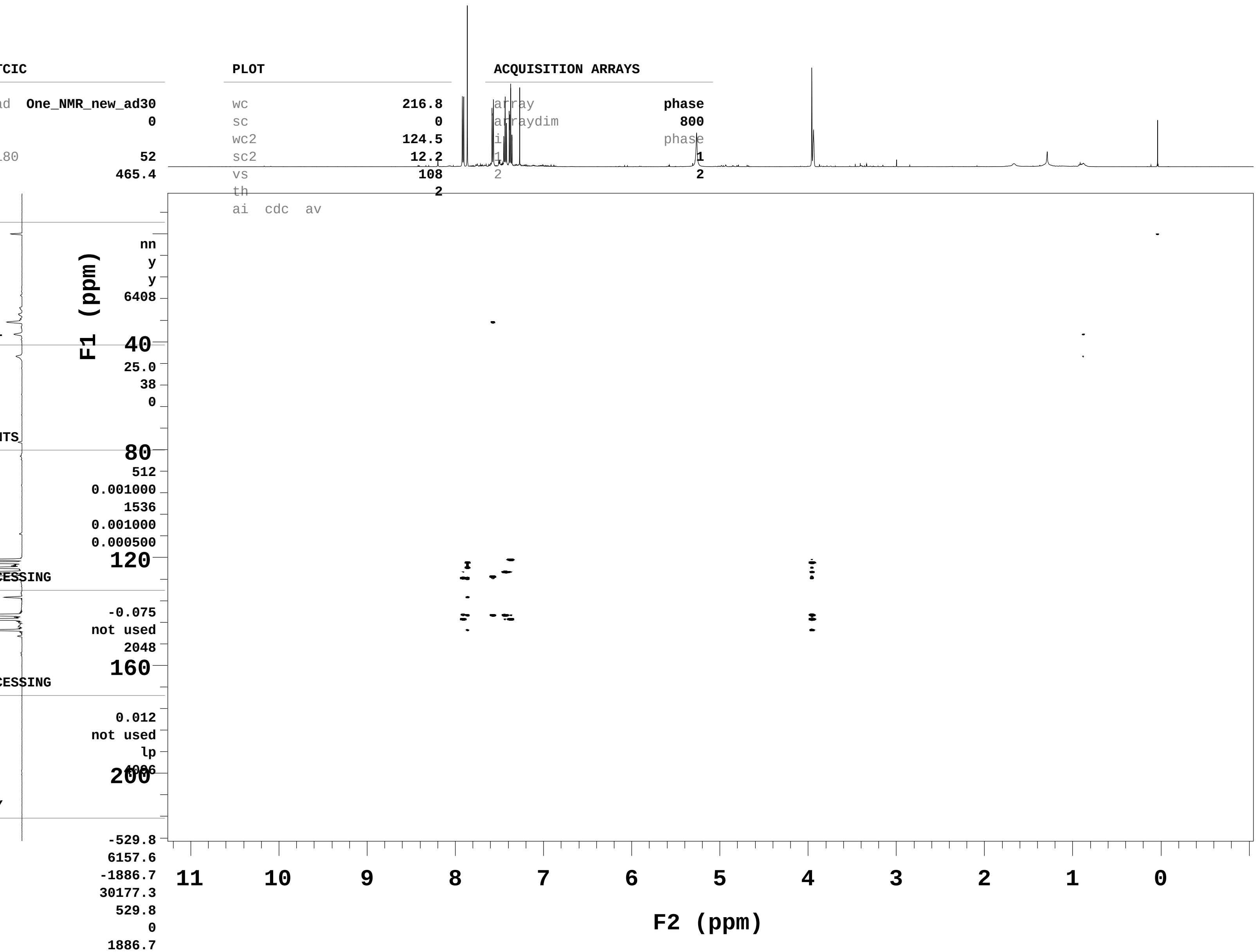

Plotname: RC-2-C-5-158-F30-39_gHMBCAD_20240913_01_plot01

Data file /home/data/MBaumann/newucd500/RuairiCrawford/2024/20240913_RC-2-C-5-158-F30-39_01/RC-2-C-5-158-F30-39_gHMBCAD_20240913_01.fid

Plot date 2024-09-13
